# Metastasis of Ciliary Body Melanoma to the Contralateral Eye: A Case Report and Review of Uveal Melanoma Literature

**DOI:** 10.1155/2015/427163

**Published:** 2015-03-22

**Authors:** Nouritza M. Torossian, Roy T. Wallace, Wen-Jen Hwu, Agop Y. Bedikian

**Affiliations:** ^1^Institut Universitaire du Cancer de Toulouse Oncopole, Toulouse, France; ^2^Retina Consultants of Nashville, Nashville, TN, USA; ^3^MD Anderson Cancer Center, Houston, TX, USA

## Abstract

Many types of cancers metastasize to the eye. However, uveal melanoma metastasizing to the contralateral choroid is very rare. We report the case of a 68-year-old man with history of treated uveal melanoma of the right eye that developed metastasis to the liver and the choroid of the left eye. Ten years earlier, he was diagnosed to have uveal melanoma of the right eye and was initially treated with plaque radiotherapy. Two years later, upon progression of the disease in the right eye he had enucleation of the eye. We describe the patient's clinical history, the diagnosis of recurrent disease in the contralateral eye, therapy of the left eye, and systemic metastasis. In addition, we reviewed the published medical literature and described the recent advances in the management of uveal melanoma.

## 1. Introduction

Uveal melanoma is the most common primary intraocular malignant tumor in adults [1]. After cutaneous melanoma, it is the second most common type of primary melanoma, representing about 3% of all melanoma diagnoses [2]. The mean incidence of uveal melanoma in the United States is approximately 5 new cases per million people [2]. There are 4100 new cases of uveal melanoma diagnosed in the United States each year [2]. The peak incidence of uveal melanoma occurs from 65 to 70 years [3]. About 98.8% of the cases occur in Caucasians. Primary uveal melanoma arises from melanocytes located in the uveal tract, which comprises the iris, the ciliary body, and the choroid of the eye [3]. Melanomas of the posterior uveal tract including the ciliary body which usually are diagnosed late in view of location generally are more aggressive, metastasize often, and have the worst prognosis [3, 4]. Symptoms related to locally advanced uveal melanoma depend on the location of the primary. Over 95% of patients have disease limited to the eye at diagnosis [5]. Currently, patients with uveal melanoma often receive eye-preserving plaque radiation therapy and enjoy the same survival as those who undergo enucleation of the eye. Approximately 40% to 50% of patients with primary uveal melanoma ultimately develop systemic metastases [6]. The 5-year and 15-year survival rates from diagnosis of uveal melanoma are 45% and 30%, respectively [6]. Of the patients who die of uveal melanoma, about 60% and 90% do so within 5 and 15 years, respectively [6].

The survival of patients with metastatic uveal melanoma is directly related to the site of metastasis. The liver is involved in as many as 95% of individuals who develop metastatic disease [7, 8] and is the exclusive site of systemic metastasis in about 40% of patients [7]. Other common metastatic sites involved include lung (46%), bone (29%), and skin (17%) [7, 8]. The median survival of patients who develop liver metastasis is reported to be 6 to 7 months, and the 1-year survival rate is estimated to be 10% to 15% [8, 9].

Little progress has been made in the management of systemic metastases [6, 9]. Local, regional and systemic therapies for metastatic uveal melanoma have had little impact on duration of patient survival [9]. Metastatic cancers including melanoma have been reported to spread to the orbit of the eye [10, 11]. Malignancies other than melanoma metastasize to the uveal tract more often. Breast cancer is the most common tumor type to metastasize to the uveal tract and comprise 47% of the cases [12]. Uveal melanoma metastatic to uveal tract of contralateral eye is very rare. Here, we report the case of a 68-year-old man with uveal melanoma of the right eye with metastasis to the choroid of the left eye 10 years after initial treatment of the right eye.

## 2. Clinical Case

The patient is a 68-year-old white male who was diagnosed to have uveal melanoma of the right eye in June 1995 when he was 58 years old. The primary tumor was a ciliary body and superior choroidal melanoma of the right eye; it was 8 mm in height and 13-14 mm in basal diameter with an early area of neovascularization of the iris. He was treated with Iodine-125 plaque radiotherapy starting on 06/13/1995. It delivered 61.2 Gy at 8 mm depth. Regression of the lesion was observed subsequently. He remained free of the disease for 2 years, and in 1997 he had pain in the right eye due to tumor regrowth. He was treated with enucleation of the eye. In December 2004, he palpated a nodule on the left neck. A fine needle aspirate (FNA) taken from left supraclavicular lymph node on 12/14/2004 showed malignant cells, but definitive diagnosis of melanoma could not be made.

Radiologic staging with CT scan of the chest, abdomen, and pelvis performed in December 2004 showed hepatic metastases. PET scan performed on 12/03/04 detected enlarged lymph nodes in the retroperitoneum, mesenteric area, and the left supraclavicular area in addition to the liver metastases. A CT guided biopsy of the liver metastasis performed on 01/03/2005 showed metastatic melanoma with cells positive for HMB-45 and Melan-A immunohistochemical stains. Review of the pathology slides of the specimens taken from the lymph node and the liver in our institution confirmed the diagnosis of metastatic melanoma. FNA from the left supraclavicular lymph node showed similar findings.

**Figure 1:**
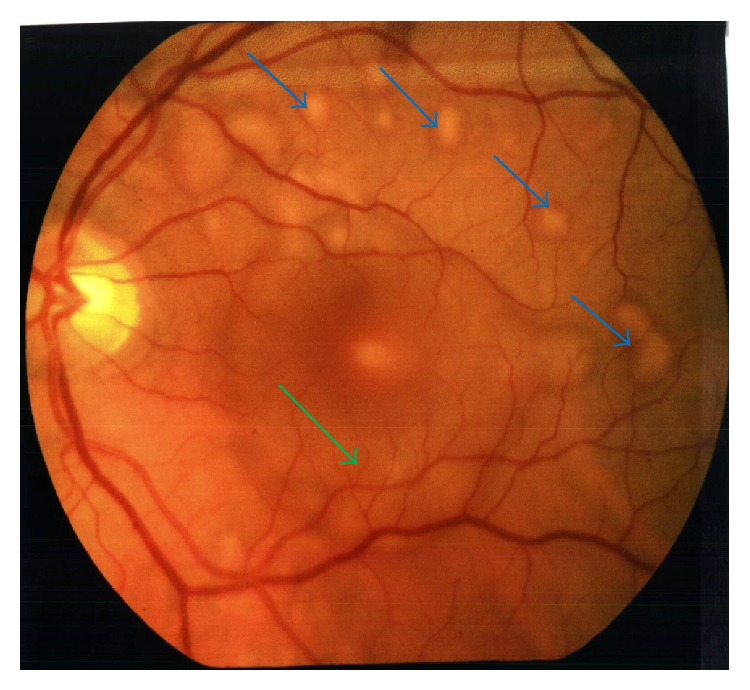
Photograph of the left eye showing creamy yellow choroidal infiltrates with subretinal fluid (blue arrows) extending through the macula (green arrow).

**Figure 2:**
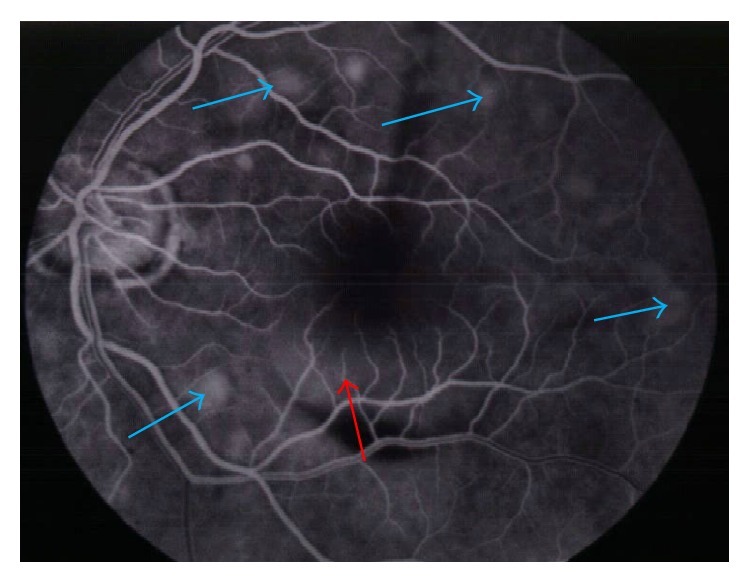
Fluorescein angiogram showing leakage of fluorescein (blue arrows) consistent with areas of choroidal infiltration seen on color photographs and late leakage with subretinal fluid (red arrow).

**Figure 3:**
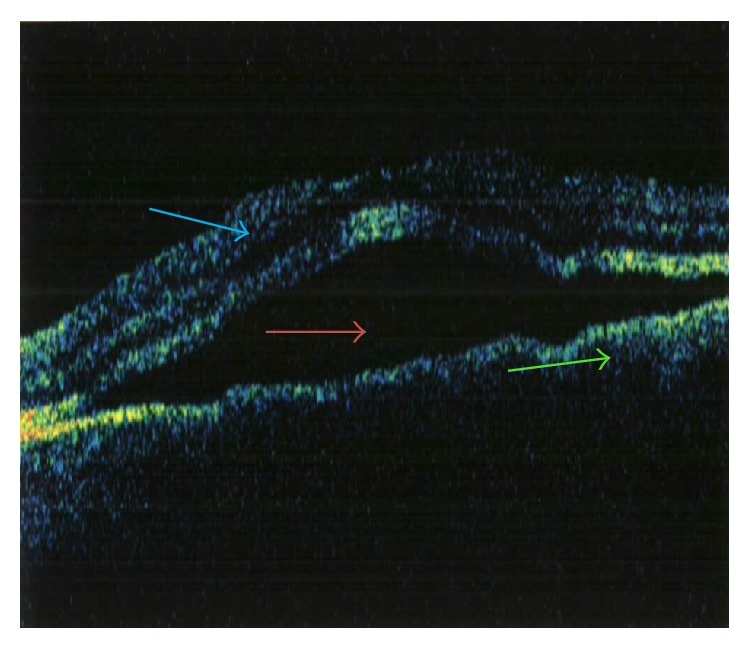
Cross-sectional optical coherence tomogram showing cross-sectional image of macula with the retina (blue arrow), subretinal fluid (red arrow), and retinal pigment epithelium and choroid (green arrow).

On 01/11/2005 the patient returned with decreased vision in his left eye of 1 week duration. He also complained of seeing black spots and floaters. An MRI of the brain performed on 01/12/2005 showed no evidence of intracranial metastases. On slit lamp fundoscopic examination, multiple small choroidal metastatic lesions were seen in the posterior pole/fundus of the left eye, each measuring about 300 to 500 microns in size, associated with an exudative retinal detachment centrally (Figure 1). Fluorescein angiogram (Figure 2) and Optical Coherence Tomography (Figure 3) confirmed presence of choroidal infiltration with melanoma. The patient's left eye was treated with plaque radiotherapy for palliation. A total activity of 46.67 millicuries was delivered using Iodine-125 plaque that was placed on 01/26/05. Dose was downgraded to 70 Gy from the typical 85 Gy and delivered quickly (in 47 hours) as this was a palliative attempt.

The patient came to M. D. Anderson Cancer Center for a second opinion after seeking advice for therapy at Vanderbilt medical center and Memorial Sloan-Kettering Cancer Center. He was seen in the clinic on 02/08/05. At the time he complained of blurry vision at the left eye in addition to abdominal pain. His vital signs were temperature 36.5, pulse 84, respiratory rate 16, and blood pressure 130/86. His Karnofsky performance status was 90%, height 164.5 cm, and weight 84.5 kg. The physical examination findings included right eye prosthesis. Left eye had clear sclera. Extraocular muscles were intact. A 2 cm left supraclavicular lymph node was palpated. Abdomen was soft and obese with the liver edge palpable at 6 cm at the midclavicular line below the costal margin. There were no other palpable abdominal masses. The rest of the physical examination was unremarkable.

The complete blood count was unremarkable. The liver profile and chemistries were normal except for the alkaline phosphatase (210, normal range 38–126 IU/L), ALT (58, normal range 7–56 IU/L), and LDH (1,477, normal range 313–618 IU/L), which were elevated. After radiologic restaging, he was started on temozolomide at 75 mg/m^2^/day × 42 days every 8 weeks. Concurrent intrahepatic chemoembolization with cisplatin, doxorubicin, and 300–500 micron PVA particles was done on 2/8/2005 for the treatment of extensive disease in the liver.

A second procedure was performed 4 weeks later. Radiologic restaging on 6/13/2005 showed progression of disease in the liver and at the extrahepatic sites. The patient's systemic therapy was changed to temozolomide, vinblastine, and cisplatin combination. Fundoscopic evaluation on 8/19/2005 showed no active choroidal metastasis. He was stable postplaque radiotherapy of his left eye. The patient expired on 12/18/2005 in a context of systemic progression.

## 3. Discussion

Only a few cases of uveal melanoma metastasis to the contralateral choroid have been reported [12–17]. In the 11 cases of skin melanoma metastasized to the uveal tract, the metastases were located in the choroid (5 cases), the iris (4 cases), and the ciliary body (3 cases) [12]. Zografos et al. [18] reported on 20 cases of melanoma metastatic to the eye and the orbit. In fifteen patients with intraocular metastasis the metastases were located in the choroid in 11 cases, in the iris and ciliary body in 2 cases, and in the retina and the vitreous in 2 cases. The primary tumors in the 14 cases of intraocular metastasis were skin melanomas (8 cases), melanomas of the contralateral eye (3 cases), mucosal melanoma (1 case), and melanomas of unknown location (2 cases). The median survival time from diagnosis of uveal tract metastasis was 8.8 (range 1–48) months [18].

In the Collaborative Ocular Melanoma Study (COMS) of primary uveal melanoma only 3 (<1%) of 739 patients with uveal melanoma had metastasis to the contralateral eye or orbit [19]. Case reports of uveal melanoma metastatic to the choroid of contralateral eye are listed in Table 1. Only one of the 7 patients listed in Table 1 did not have liver metastasis. Three patients were found to have metastasis to the choroid of the contralateral eye within 5 years from diagnosis of primary uveal melanoma. Three additional patients were diagnosed to have metastasis to contralateral choroid 10 or more (range 10–17) years after diagnosis of the primary.

In addition to metastasis to the contralateral eye, our case had several uncommon features. The patient's primary melanoma was located in the ciliary body, which is the primary site in only 12% of uveal melanoma cases. The patient also had retinal detachment, which is uncommon at presentation of melanoma of the ciliary body [20]. While most patients with metastatic uveal melanoma present with symptoms related to liver metastasis, our patient presented with left neck lymph node metastasis as the first sign of tumor recurrence. Metastasis to a neck lymph node is very rare and occurs primarily in patients who have invasion of the conjunctiva by the primary tumor. Only 2–4% of patients with advanced uveal melanoma develop lymph node metastasis, and most often the affected lymph nodes are located at the porta hepatis and/or mesentery [6, 21, 22].

While the median time to systemic tumor recurrence in our previously published review of uveal melanoma cases was 3.25 years [21], the patient whose case is described here was diagnosed with metastasis to the contralateral eye 10 years after treatment of the primary tumor, similarly to several other patients with metastasis to choroid of contralateral eye. A detailed ophthalmoscopic examination by an eye specialist confirmed the presence of multiple choroidal metastases. These findings were confirmed with ultrasonography and angiography. Although we did not biopsy the tumor in the choroid of the contralateral eye to confirm tumor metastasis, in view of concurrent histologically confirmed melanoma metastases in the left supraclavicular lymph node and the liver, the presence of left choroidal metastasis was highly likely.

Plaque radiotherapy significantly alleviated the patient's symptoms related to his recurrent tumor in the contralateral eye and preserved his vision. Lack of control of liver metastasis is associated with poor prognosis [6, 9, 22]; chemoembolization using doxorubicin, cisplatin, and polyvinyl alcohol particles was administered. Unfortunately, the patient's tumor progressed, resulting in the patient's death.

Over the past decade, significant progress has been made in identifying patients at high risk for tumor recurrence and in the use of targeted therapies for management of systemic metastasis based on tumor molecular markers. It has been well established that monosomy 3 is associated with significantly higher risk for systemic metastasis and shorter survival [23, 24]. Certain genes have been identified to be predictive of metastasis in uveal melanoma. A 15-gene microarray assay, which includes 3 control genes, has been successful in discriminating between low-grade and high-grade (class 2) uveal melanomas [25–27]. The multigene expression assay has become a useful tool for selecting patients at high risk for tumor recurrence that may benefit from future adjuvant trials. Inactivation of the* BAP1 *gene which codes for an enzyme that binds to BRCA1 and BARD1 in regulating the tumor suppressor complex has been described in up to 84% of class 2 uveal melanomas [28, 29]. Loss of BAP1, as is the case in monosomy 3, may predispose a patient to uveal melanoma for tumor recurrence. In addition, identification of specific genes causing downregulation of tumor suppressor genes on chromosome 3 and upregulation of genes on chromosome 8q has improved our ability to use those genes as potential targets for control of metastatic disease [25–27]. Each of* GNAQ *and* GNA11 *has been found in 40% to 45% of patients with metastatic uveal melanoma and, together with several other genes, is associated with systemic spread of uveal melanoma cells [24, 30, 31].* GNAQ *and* GNA11 *mutations cause upregulation of the MAPK pathway, and responses to the MEK inhibitor selumetinib have been reported [32, 33] in early studies. A large study investigating the use of selumetinib for treatment of uveal melanoma is currently in progress.

## Figures and Tables

**Table 1:** Case reports of uveal melanoma metastatic to the contralateral eye.

Reference	Age in years/sex	Site of primary tumor; therapy	Time^a^ to contralateral eye metastasis	Site(s) of extraocular metastasis
Li et al. [13]	75/female	Left eye: enucleation	2 years	Lung, liver
C. L. Shields and J. A. Shields [12]	29/female	Right eye; cobalt 60 plaque radiotherapy, enucleation 9 years later	17 years	Liver, lung, brain
Mitjana et al. [14]	53/male	Left eye	1 year	Skin
Singh et al. [15]	49/female	Left eye; ruthenium 106 plaque radiotherapy	15 years	Liver
Khetan et al. [16]	52/male	Left eye; enucleation	74 months	Abdomen, liver
Shields et al. [17]	52/male	Left eye; enucleation	52 months	Orbit, eyelid, liver
This report	58/male	Right eye; plaque radiotherapy, enucleation 2 years later	10 years	Left cervical, superclavicular, retroperitoneal and mesenteric lymph nodes, liver

^a^From initial presentation.
